# Doing Experimental Psychological Research from Remote: How Alerting Differently Impacts Online vs. Lab Setting

**DOI:** 10.3390/brainsci12081061

**Published:** 2022-08-10

**Authors:** Fiorella Del Popolo Cristaldi, Umberto Granziol, Irene Bariletti, Giovanni Mento

**Affiliations:** 1 Department of General Psychology, University of Padova, Via Venezia 8, 35131 Padova, Italy; 2 Padova Neuroscience Center, University of Padova, Via Orus 2/B, 35129 Padova, Italy

**Keywords:** experimental psychology, online data collection, dynamic temporal prediction task, alerting

## Abstract

Due to pandemic-imposed restrictions on lab-based research, we have recently witnessed a flourishing of online studies in experimental psychology, based on the collection of fine behavioral measures such as reaction times (RTs) and accuracy. However, it remains unclear whether participants’ alerting levels may have a different impact on behavioral performance in the online vs. lab setting. In this work we administered online and in-lab the dynamic temporal prediction (DTP) task, which requires an implicit modulation of participants’ alerting by alternating experimental conditions implying either slower or faster response rates. We then compared data distribution, RTs, accuracy, and time-on-task effects across the adult lifespan between the settings. We replicated online and across the whole age range considered (19–69 y) all the task-specific effects already found in-lab (both in terms of RTs and accuracy) beyond the overall RTs delay typical of the online setting. Moreover, we found an interaction between the setting and task-specific features so that participants showed slower RTs only in experimental conditions implying a less urgent response rate, while no RTs delay and a slight accuracy increase emerged in faster conditions. Thus, the online setting has been shown to be methodologically sound in eliciting comparable effects to those found in-lab. Moreover, behavioral performance seems to be more sensitive to task-induced alerting shifts in the online as compared to the lab setting, leading to either a heightened or reduced efficiency depending on a faster or slower response rate of experimental conditions, respectively.

## 1. Introduction

Experimental psychology has traditionally used a structured methodology for data collection, based on a strict control of the laboratory setting [1]. This approach implied the implementation of different phases, such as the conceptualization of the study, the formulation of hypotheses, the participants’ recruitment procedures, the control of laboratory’s environmental characteristics (e.g., brightness, temperature, humidity, quietness), and the use of techniques and tools ensuring high-precision spatial and temporal control of stimuli presentation [1]. Altogether, these procedures provided experimental psychology with a sound epistemological foundation, making it a reliable scientific discipline [2]. With the advent of computers and information technology, the degree of precision in behavioral data collection advanced even further. In particular, thanks to software dedicated to behavioral measures’ recording [3], it was possible to automate data collection procedures, reaching a finer experimental control.

Crucially, although lab-based research has ensured for decades reliable data quality and the possibility to replicate results by sharing experimental protocols between researchers and labs, it inevitably clashed with some practical aspects that can make its implementation difficult. First, the need to have a physical laboratory facility equipped with constantly updated devices and software for data collection and able to ensure standardized environmental conditions. This could imply logistical difficulties when large samples are required, and a prolonged use of the lab space, which is often shared between several researchers, is needed. Second, a physical limit is necessarily imposed by sequential data collection, i.e., when behavioral measures are collected from a single participant at a time. Given the need to build large datasets to increase experiments’ reliability and statistical power, in accordance with the guidelines recently proposed by the scientific community (see Open Science Framework initiative, OSF https://osf.io, accessed on 7 July 2022), researchers are often called to make choices. On the one hand, the increasing pressure to enlarge the number of publications per year pushes researchers to collect, analyze, and publish results in the shortest time possible. On the other hand, large sample sizes are increasingly required. Yet, *a priori* G*power calculations may be insufficient especially when multiple-level interactions are analyzed. This implies the risk of negatively affecting data quality in the attempt to reconcile speed of data collection with large sample sizes, consequently threatening results’ replicability, especially for early-career researchers (i.e., who are pushed by the incentive system to the maximum quantitative productivity) [4]. Online data collection was proposed as a possible solution to address these issues [5,6], and evidence on its advantages exponentially grew in recent years (for a discussion, see [6,7,8,9,10]). Transferring the experimental setting to the web could allow researchers to effectively reach and test large numbers of individuals from around the world [11]. The online setting offers indeed both *efficiency*, given the ease, speed, and cost-effectiveness of collecting accurate data [12,13], and *accessibility*, given the possibility of reaching samples otherwise difficult to recruit [14,15,16,17,18]. Last but not least, the possibility to collect large amount of data through online methods improves the *generalizability* of results.

While running online experiments has long represented a valuable possibility for psychologists interested in collecting large datasets in a short time, the last years of the COVID-19 pandemic and the resulting lockdown of lab facilities forced the researchers carrying lab-based research to adapt their experimental protocols to the online setting, moving de facto from seeing this methodology as an opportunity to seeing it as a necessity [19]. Consequently, we have recently witnessed a flourishing of online studies based not only on the collection of questionnaires and surveys but also on finer measures such as reaction times (RTs) and accuracy of behavioral responses. In this rapidly evolving scenario, experimental studies investigating the comparability between the online and lab settings become particularly interesting for the scientific community, especially in view of the considerable variability derived by the use of different hardware and software components between participants in the online setting. Hardware components include, for example, computer devices (e.g., PC, Mac, Linux, tablet, cellphones, etc.) with different data processing capabilities (e.g., CPU, RAM, audio-video card, etc.), which may lead to non-standardized physical features (e.g., brightness, contrast, loudness, screen size) and thus to a huge variability in stimuli presentation and variations in timing of stimuli and response. As software components, different platforms for the creation of experimental protocols (e.g., experiment builders such as OSWeb, Pavlovia), for participants’ recruitment (e.g., Prolific, Amazon’s MTurk), and for experiments’ hosting (e.g., JATOS, Gorilla) may add up with human factors (e.g., instructions delivering and comprehension, performance feedback or control, etc.) in increasing researchers’ degrees of freedom when designing online experiments [20].

Despite the potentially biasing factors of the online setting (thoroughly reviewed in a recent paper by [20]), carefully developed online studies still have a huge potential for methodological soundness. Specifically, experimental protocols requiring a not excessively tight temporal resolution of stimulus delivering and response collection appear particularly suitable for online studies [20]. In contrast, experimental paradigms extremely sensitive to the temporal sequencing of stimuli (i.e., with less than 50 ms of Stimulus Onset Asynchrony—SOA), such as attentional blink or masked-priming tasks, are not ideally suited for online data collection [21,22]. Nonetheless, several time-sensitive experimental effects, such as the Stroop effect or the above-mentioned attentional blink and masked-priming effects, have been replicated online [23].

Besides the peculiarities of the single tasks, studies comparing the lab setting with the online one consistently found that mean response speed is systematically delayed in online experiments, with a reported delay range between 25 and 60 ms [22,24,25,26]. This systematic delay is an intrinsic, unavoidable technical limit of online research most likely due to the variability in browsers/operating systems of participants’ personal computers [3,22]. Nevertheless, online tools show a reasonable overall temporal accuracy since the delay is reflected in the absolute RTs measures, and it appears constant within the same software–browser–operating system combination [3]. Most importantly, regardless of the absolute RTs delay, the magnitude of experimental effects within several cognitive tasks (e.g., decision-making tasks, double tasks, facial expression recognition tasks, lexical decision tasks, natural language generation) seems to be comparable between the online and laboratory settings [27,28,29,30]. In sum, although an online implementation may lead to potential noise factors, there is consensus that online research provides researchers with an effective means for collecting sound behavioral data [3,20,31,32]. In addition to this, the evident savings in terms of time and money, combined with the possibility of collecting large datasets, seem to largely compensate for the potential negative aspects of this methodological approach [20].

Notwithstanding, some open questions about the comparability between online and lab-based research in psychology still remain unaddressed. For example, although online data collection could represent a useful solution to overcome many lab-based research limitations, it imposes a major concern regarding sample representativeness [33]. In addition, a cogent question regards whether online data collection can impact differently on the alerting state of participants, biasing their behavioral performance. Indeed, remote execution does not allow for a strict time-by-time control of people’s response speed and accuracy. This drawback can be partially mitigated by providing participants with either some reward (e.g., money or course credits) or feedback on their task performance [21]. Yet, the physical absence of the experimenter and the consequent unbiased social desirability and low task-related motivation of participants could negatively impact on experiments’ execution [20,33,34]. Those aspects could especially influence tasks involving a large number of trials and implying repetitive and fast responses, which could induce a block-wise decrease in response speed and/or accuracy. Therefore, better understanding of whether performance shifts during the task (namely, time-on-task effects [35,36]) are negatively impacted in the online setting clearly emerges as one of the core issues for advancing psychological research.

Given the importance of time-on-task effects as potentially biasing factors, the aim of the present study was to examine across the adult lifespan whether and to what extent tasks based on a modulation of participants’ alerting and attention at an *implicit* level, such as the dynamic temporal prediction (DTP) task [37], could elicit comparable experimental effects in the online vs. laboratory setting. The ability to automatically and implicitly detect statistical regularities in the environment is in fact a fundamental aspect of human cognition, and it plays an important role in shaping behavior, motor preparedness, perception, and cognitive functions in general [38,39,40]. Thus, targeting implicit tasks when comparing the online with the lab setting as well as considering the whole adult lifespan may offer a precious contribution to both the theoretical and methodological levels.

To this purpose, we administered online the DTP task [34] to an adult sample aged 19–69 years, and we compared the data collected online with a dataset previously acquired in the laboratory with the same task. The DTP task consists of a brief, computerized detection task collecting simple RTs to warned, visual stimuli. In the DTP task, a warning stimulus (S1) is followed by the presentation of an imperative stimulus (S2), to which participants must respond as fast and accurately as possible. The task investigates the flexibility of motor control by inducing implicit temporal expectancy at both the trial- (local) and the block-wise (global) level. More specifically, the effect of the local predictive rules on behavioral performance is investigated by employing three different trial-by-trial SOA intervals (short: 500 ms; medium: 1000 ms; long: 1500 ms), whereas the effect of the global predictive rules is investigated through the block-wise manipulation of three different probability distributions per each SOA, yielding to fast blocks (prevalence of short SOA intervals), uniform blocks (three SOA intervals equally distributed), and slow blocks (prevalence of long SOA intervals). Moreover, the DTP task allows to obtain an index of the implicit adaptation of motor response to global predictive rules (delta score) by calculating the difference in RTs between slow and fast blocks. Importantly, participants are not explicitly instructed about the different predictive rules involved in the paradigm: this allows to study participants’ ability to implicitly adjust performance speed and accuracy as a function of either local or global predictive rules. Lastly, this paradigm requires a high-sensitive (but not extreme) stimuli delivery timing, preventing it from being inadequate to the online setting [21,22]. These characteristics make the DTP task particularly suitable for the purposes of our investigation, namely comparing data distribution, RTs, accuracy, and time-on-task experimental effects between the online and lab settings.

In line with the literature, we hypothesized to find (H1a) slower RTs in the online vs. lab setting [3,22] and (H1b) no significant differences in performance accuracy between the two settings [22]. We also expected to replicate in the online setting the effects of the paradigm previously found in the lab: (H2a) the local prediction effect, with faster RTs and lower accuracy in trials with long vs. medium and short SOA [34,35,36,37]; (H2b) the global prediction effect, with faster RTs in fast blocks and slower RTs in slow blocks as compared to the uniform block [34,35,36,38]; and (H2c) the implicit learning effect, reflected by a positive delta score between slow and fast blocks [34,36]. Moreover, since the DTP task implicitly induces response speed changes between the blocks, it could be possible to find (H3) an interaction between block and setting (online vs. lab) with potentially slower RTs in the online setting especially in less arousing blocks (uniform, slow). Lastly, we expected (H4) that in both settings, the adaptation of response speed to local–global changes in the task was affected by age, with a progressive loss of efficiency in flexible adaptive motor control as age increased.

## 2. Materials and Methods

### 2.1. Participants

A total of 255 volunteer participants (78 males, age: M = 40.68, SD = 17.7, range = 19–69) took part in the experiment either online or in the lab setting. They were enrolled via social media (e.g., Facebook) or through university courses, and all signed a written consensus (lab group) or agreed to participate by clicking a link (online group) after receiving information about experimental procedure and data treatment. The study was approved by the Ethical Committee for the Psychological Research of the University of Padua (protocol no. 3666) and was conducted in accordance with the Declaration of Helsinki. Participants were free to withdraw at any time by closing the browser window in the online setting or by leaving the room in the lab setting. For each participant, demographic information (age, gender) was collected (see Table 1). The two groups (online vs. lab) were slightly unbalanced for gender and age.

Before the task, inclusion criteria for participation were assessed. All participants must report having normal or corrected-to-normal vision, no neurological and/or psychiatric disorders, and no drugs or psychoactive substances use. Participants over 60 years of age with cognitive difficulties, i.e., a score below 25 in the Mini Mental State Examination (MMSE) [39,40] for the lab setting and a score of 8 or below in the 10-item Short Portable Mental Status Questionnaire (SPMSQ) [41] for the online setting, were excluded from participation. Despite being different, the MMSE and the SPMSQ are both acknowledged in the literature as reliable tools to assess cognitive functioning in aging, providing comparable results [42]. Since the MMSE cannot be administered remotely, we employed the SPMSQ for the online setting.

### 2.2. Experimental Procedure

Data collection occurred in two different settings: online on participants’ personal computers at a quiet location of participants’ choice and in the laboratory. The online study was run through OpenSesame [43] and the JATOS hosting server [44], both open-source web platforms for online studies. The lab study was run using E-Prime 2 software (Psychology Software Tools, Pittsburgh, PA, USA [45]). In the lab setting, stimuli were presented on a laptop with a 15-inch monitor at a resolution of 1280 × 1024 pixels. Participants were seated comfortably in a chair at a viewing distance of around 60 cm from the monitor. All participants performed the DTP task [34].

**Figure 1:**
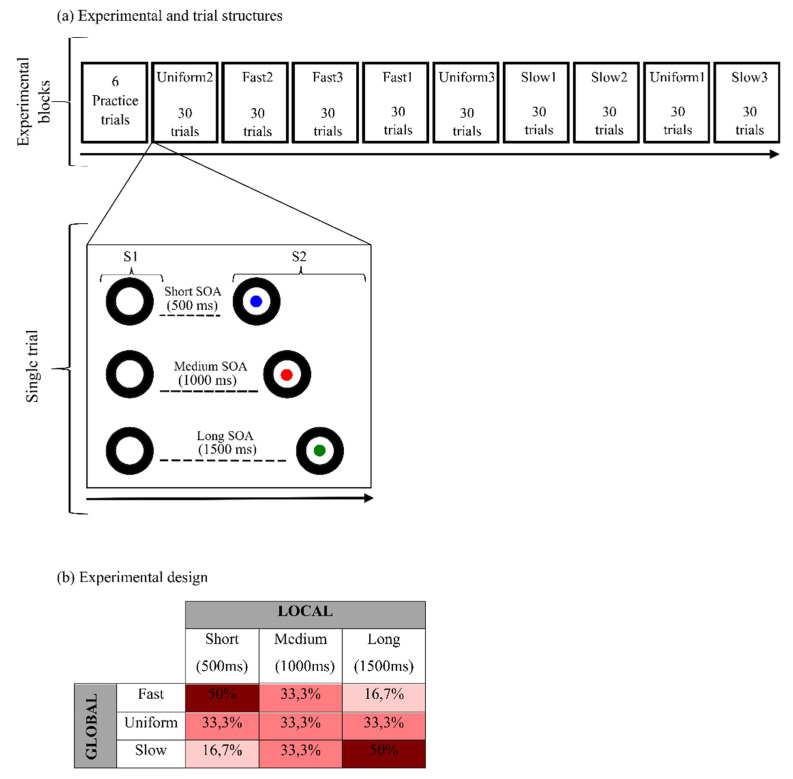
Dynamic temporal prediction (DTP) task. Experimental procedure included 1 practice block and 9 test blocks. Blocks could be uniform, fast, or slow. Each block was randomly administered 3 times. The figure shows (**a**) an example of block order. Each block included 30 trials, for a total of 270 trials. The single trial structure is illustrated: S1 (cue/black circle) can be followed by a short (500 ms), medium (1000 ms), or long (1500 ms) SOA before S2 occurrence (target/cartoon character, here represented with colored circles for illustrative purposes due to copyright restriction). To assess the effect of global prediction, (**b**) different probabilistic distributions per each SOA (short, medium, long) were created a priori. SOA could be equally distributed (uniform), fast (biased toward the short SOA interval), or slow (biased toward the long SOA interval; adapted from [34], reproduced with permission from [34].

The experimental procedure included 1 practice block and 9 test blocks. At the beginning of the task, a block of 6 practice trials was presented. During practice, all participants received trial-by-trial feedback based on their performance. Specifically, a yellow smile was displayed if anticipatory (before target onset), premature (<150 ms from target onset), or excessively slow (>1000 ms from target onset) responses were provided, while a green smile was displayed if the RT was between 150 and 1000 ms. Then, test blocks were presented. Each block type (fast, uniform, slow; see 2.5 below for details) was administered 3 times for a total of 9 blocks and included 30 trials for a total of 270 trials (see Figure 1). SOA and block type sequence was randomized for each participant. The total length of the experiment was about 15 min. Pauses occurred about every 2 min, but no pauses were introduced between blocks to avoid participants inferring the change in the global probability distribution. Notably, participants were also left uninstructed about the presence of between-block different probabilistic distributions to ensure they did not know about global rule changes.

### 2.3. Trial Structure

Each trial began with the presentation of a warning visual stimulus (S1) followed by the display of an imperative visual stimulus (S2). S1 consisted of a picture of a black camera lens. S2 consisted of a picture of a cartoon character, which was presented centrally within the camera lens. The inter-trial interval (ITI) was randomly manipulated between 1500 and 2000 ms. Participants performed a speeded target-detection task. They were required to press the spacebar on the keyboard as quickly as possible at S2 onset (see Figure 1).

### 2.4. Local Predictive Context

To explore the effect of the local predictive context on behavioral performance, the S1–S2 SOA was varied trial-by-trial within each experimental block. Three fixed foreperiod (FP) intervals were present: short (500 ms), medium (1000 ms), or long (1500 ms). This manipulation introduced in each block three levels of temporal preparation to S2 onset, allowing us to investigate local prediction as the effect of increase of temporal expectancy as a function of SOA length on task performance. Indeed, the use of a variable S1–S2 SOA dynamically biases the subjective temporal expectancy [37,46,47,48,49]. In line with the literature [37], we expected participants to be fastest at detecting the targets appearing at the longest SOA and slowest at those occurring at the shortest SOA.

### 2.5. Global Predictive Context

To investigate the effect of the global predictive context, three different probability distributions per each SOA were created, yielding three different block types: fast (biased toward short SOA intervals), uniform, and slow (biased toward long SOA intervals; see Figure 1).

#### 2.5.1. Uniform Block

In this condition, the uniform SOA distribution yielded a medium-speed block acting as a baseline. Specifically, this consisted of a rectangular distribution of the three SOA so that the probability of each SOA in the block was equally distributed (33.3% for each SOA). The FP effect is usually expected to emerge in an a priori uniform distribution [37]. As time passes, the conditional probability of S2 occurrence increases exponentially in virtue of the fact that it has not occurred yet [37,38,47]. Consequently, motor preparedness will be lowest for short SOA and highest for long SOA.

#### 2.5.2. Fast Block

In the fast block, an a priori distribution biased toward the short SOA was present. The relative percentage was 50%, 33.3%, and 16.7% for the short, medium, and long SOA, respectively. This distribution, known as the non-aging distribution [38,50], is intended to counterbalance the increase of temporal expectancy as a function of SOA length.

#### 2.5.3. Slow Block

In the slow block, the relative percentage was 16.7%, 33.3%, and 50% for the short, medium, and long SOA, respectively. In the literature, the a priori distribution biased toward the long SOA is also known as aging distribution [38,50]. This distribution is inserted to exacerbate the increase of temporal expectancy as a function of SOA length.

### 2.6. Experimental Design and Data Analysis

The experimental design yielded a 2 × 3 × 3 factorial design, that is, *group* (between-subject: online, lab) × *SOA* (within-subjects: short, medium, long) × *block type* (within-subjects: fast, uniform, slow).

Both mean accuracy and RTs to targets were collected separately per experimental condition and per participant. Only responses between 150 ms and 1000 ms from target onset were considered as correct and included in the analysis. RTs were log-transformed in order to account for their skewed distribution [51,52]. Accuracy was computed as the percentage of correct responses over the total number of trials per condition. Delta scores were computed as the difference in RTs between slow and fast blocks.

We compared RTs and accuracy distributions between the two groups (online vs. lab) by means of both visual inspection of the *empirical cumulative distribution function* (ECDF) and paired two-sample Kolmogorov–Smirnov tests. This allowed us to explore whether data within the two groups (online vs. lab) were drawn from the same probability distribution.

In order to compare the two distributions neat of the other experimental variables (i.e., SOA, block), for each dependent variable (DV), we fitted the following *linear models (LMs)* or *(generalized) linear mixed-effects models ((G)LMMs)* with individual random intercept:

1. Log-RTs: LMM with *group* (online, lab), *SOA* (short, medium, long), *block type* (fast, uniform, slow), and their interaction as fixed factors and gender (M, F) and age as covariates;
2. Accuracy: Logistic GLMM with *group*, *SOA*, *block type,* and their interaction as fixed factors and gender and age as covariates (the percentage of correct responses was weighted on the total number of possible correct responses per each condition);
3. Delta scores: LM with *group* as predictor and gender and age as covariates.

All statistical analyses were performed through R statistical software [53]. LMMs effects were evaluated using *F*-test and *p*-values, calculated via Satterthwaite’s degrees of freedom method (α = 0.05, R package: lmerTest [54]); GLMMs effects were evaluated through Type II Analysis of Deviance (R package: car [55]); LMs effects were evaluated using *F*-test and *p*-values, calculated via Type III Analysis of Variance (R package: car [55]). For SOA and Block type variables, treatment contrasts were used, setting the long condition (i.e., long SOA and long biased block) as the reference level. For all the other variables, contrasts were set by using effect coding. Such contrast coding was applied for all the tested models. Post hoc pairwise comparisons between the levels of fixed factors were tested by means of estimated marginal means (EMMs) contrasts, Tukey adjusted for multiple comparisons (R package: emmeans [56]). For each model, we reported the estimates with standard error (*SE*), 95% confidence interval (*CI*), and the associated statistics (*t*-test for L(M)Ms, *z*-test for GLMMs). Moreover, for each LMM and GLMM, we reported the marginal and conditional R^2^ (estimated as in [57]), and for each LM, we reported adjusted R^2^.

## 3. Results

### 3.1. Descriptive Statistics

The mean RTs, accuracy (%), and delta scores per group and experimental condition are summarized in Table 2.

### 3.2. Distributions Comparison

#### 3.2.1. Reaction Times

**Figure A1:**
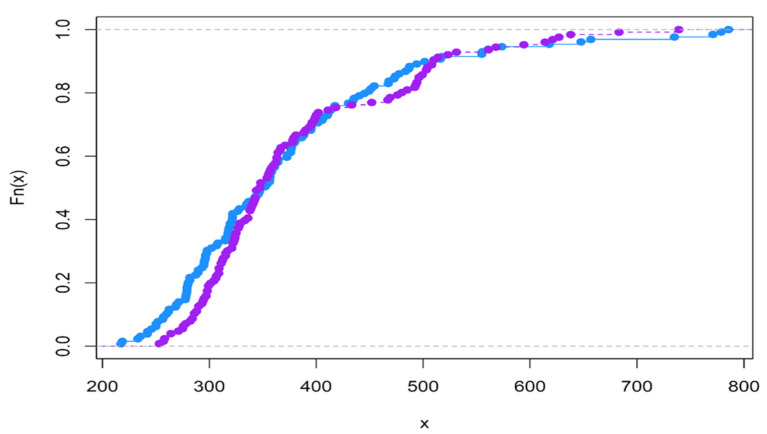
*Empirical cumulative distribution function* (ECDF) of reaction times (RTs) in the fast block–short SOA per group. *X*-axis refers to the RTs (in m); *y*-axis refers to the ECDF of RTs. Purple = online group. Blue = lab group.

**Figure A2:**
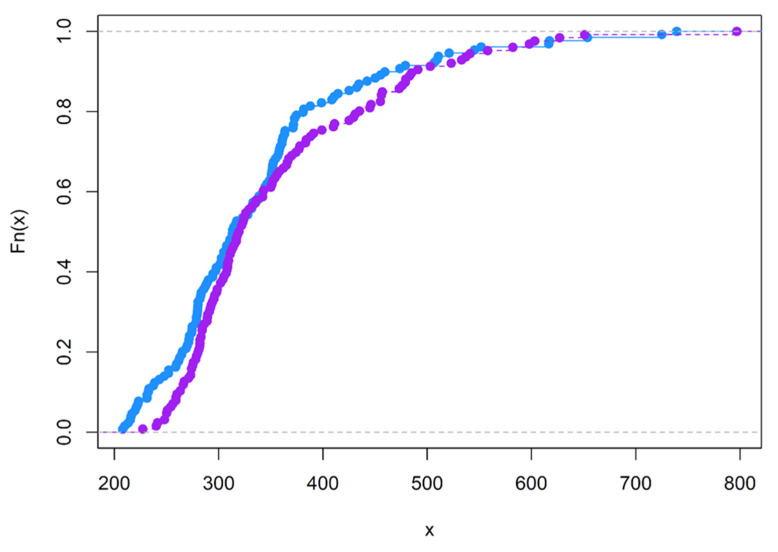
*Empirical cumulative distribution function* (ECDF) of reaction times (RTs) in the fast block–medium SOA per group. *X*-axis refers to the RTs (in m); *y*-axis refers to the ECDF of RTs. Purple = online group. Blue = lab group.

**Figure A3:**
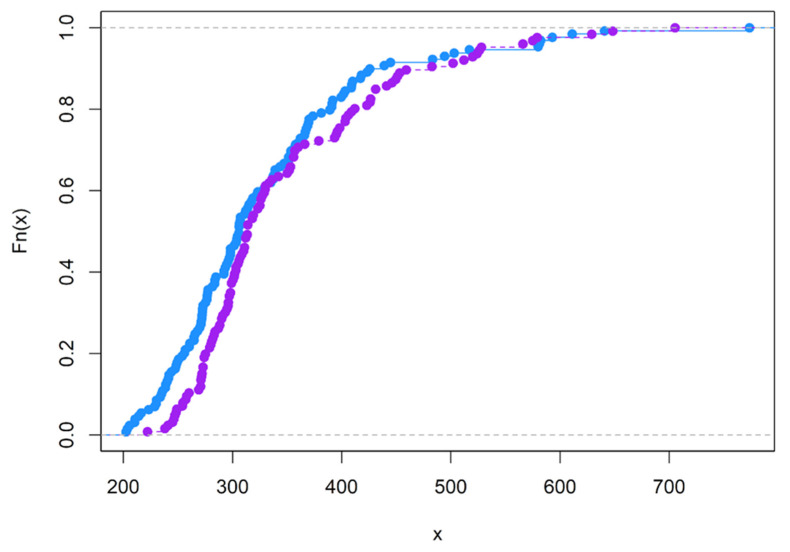
*Empirical cumulative distribution function* (ECDF) of reaction times (RTs) in the fast block–long SOA per group. *X*-axis refers to the RTs (in m); *y*-axis refers to the ECDF of RTs. Purple = online group. Blue = lab group.

**Figure A4:**
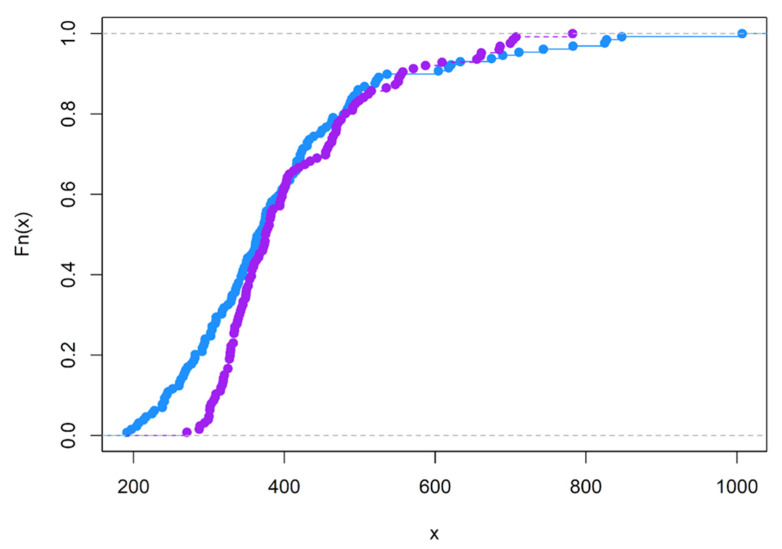
*Empirical cumulative distribution function* (ECDF) of reaction times (RTs) in the uniform block–short SOA per group. *X*-axis refers to the RTs (in m); *y*-axis refers to the ECDF of RTs. Purple = online group. Blue = lab group.

**Figure A5:**
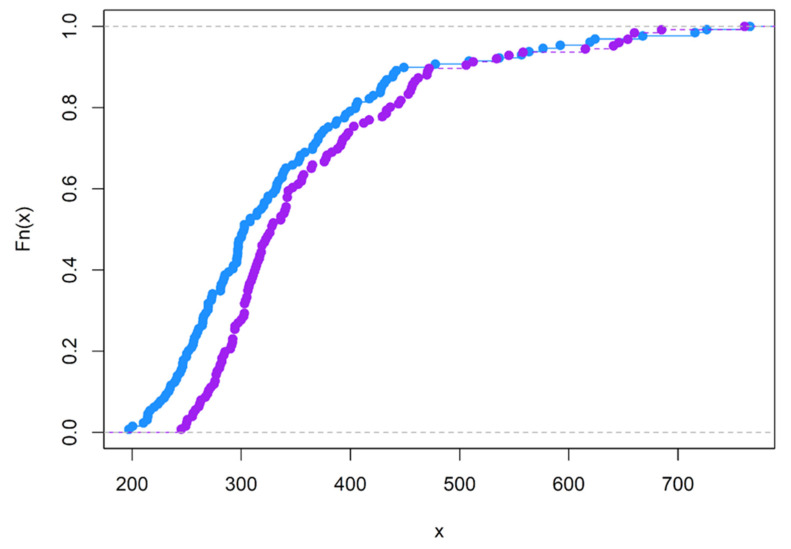
*Empirical cumulative distribution function* (ECDF) of reaction times (RTs) in the uniform block–medium SOA per group. *X*-axis refers to the RTs (in m); *y*-axis refers to the ECDF of RTs. Purple = online group. Blue = lab group.

**Figure A6:**
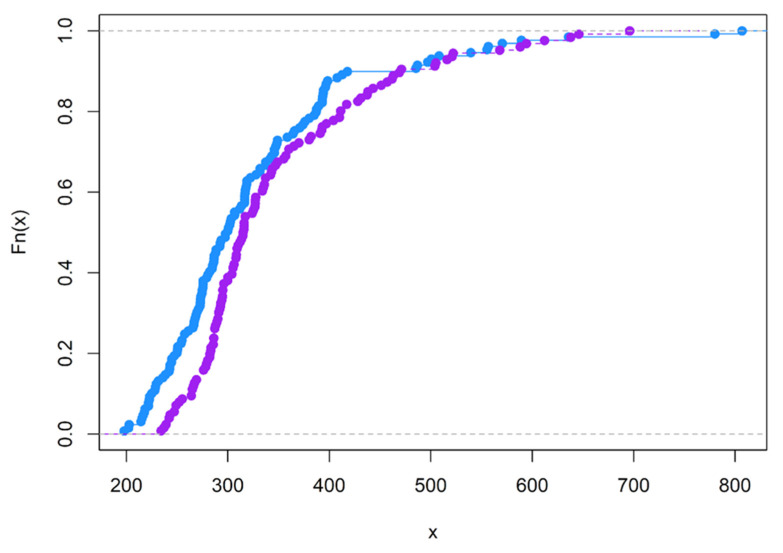
*Empirical cumulative distribution function* (ECDF) of reaction times (RTs) in the uniform block–long SOA per group. *X*-axis refers to the RTs (in m); *y*-axis refers to the ECDF of RTs. Purple = online group. Blue = lab group.

**Figure A7:**
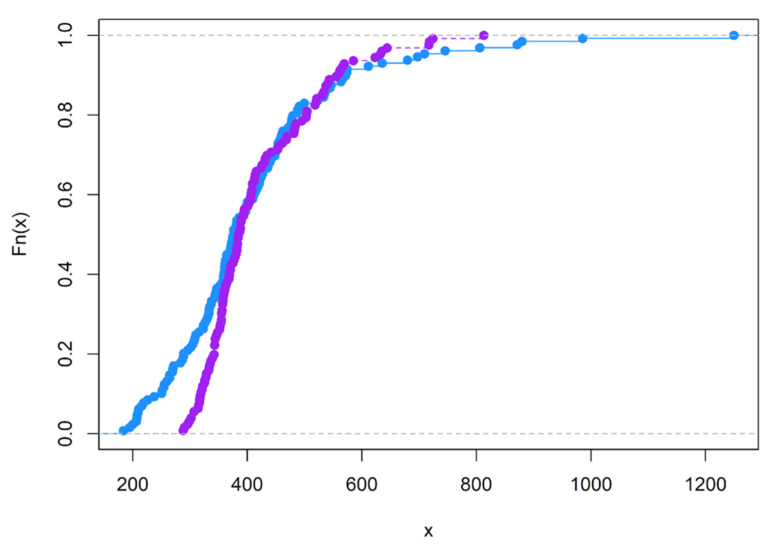
*Empirical cumulative distribution function* (ECDF) of reaction times (RTs) in the slow block–short SOA per group. *X*-axis refers to the RTs (in m); *y*-axis refers to the ECDF of RTs. Purple = online group. Blue = lab group.

**Figure A8:**
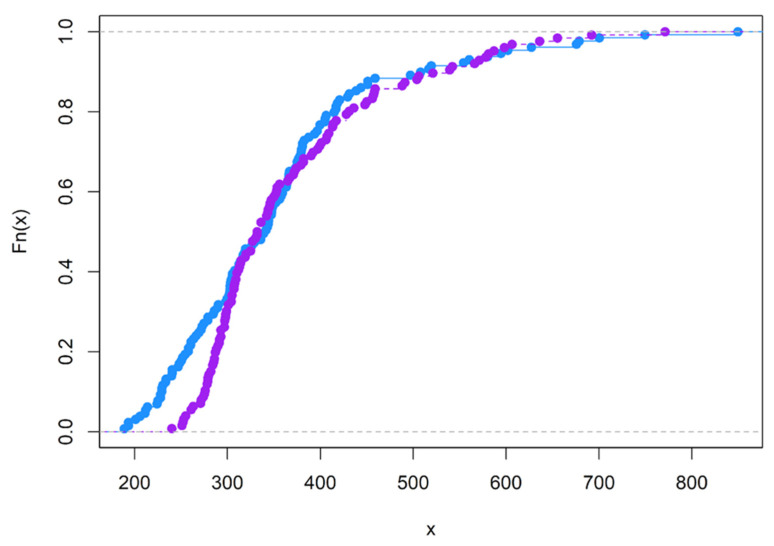
*Empirical cumulative distribution function* (ECDF) of reaction times (RTs) in the slow block–medium SOA per group. *X*-axis refers to the RTs (in m); *y*-axis refers to the ECDF of RTs. Purple = online group. Blue = lab group.

**Figure A9:**
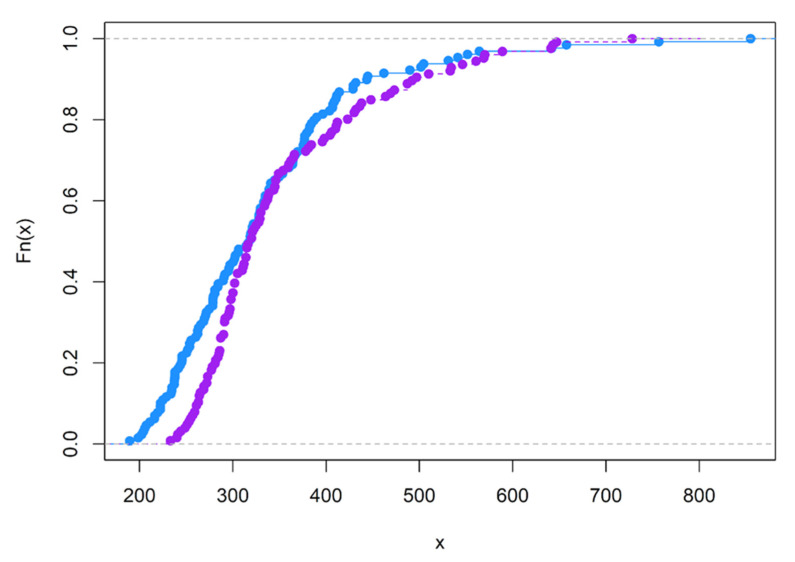
*Empirical cumulative distribution function* (ECDF) of reaction times (RTs) in the slow block–long SOA per group. *X*-axis refers to the RTs (in m); *y*-axis refers to the ECDF of RTs. Purple = online group. Blue = lab group.

Visual inspection of RTs ECDF plots (see Appendix A, Figure A1, Figure A2, Figure A3, Figure A4, Figure A5, Figure A6, Figure A7, Figure A8 and Figure A9) revealed only a partial overlap between the distribution curves of the two groups (online vs. lab) within slow and uniform blocks in all the SOA intervals (short, medium, long), whereas a greater overlap was observed within the fast blocks in all the SOA intervals. Visual inspection’s qualitative analysis is supported by the results of Kolmogorov–Smirnov test comparing RTs distributions between the two groups: statistically significant differences were found between the RTs of the two groups only in slow and uniform blocks but not in fast blocks (see Table 3).

#### 3.2.2. Accuracy

**Figure A10:**
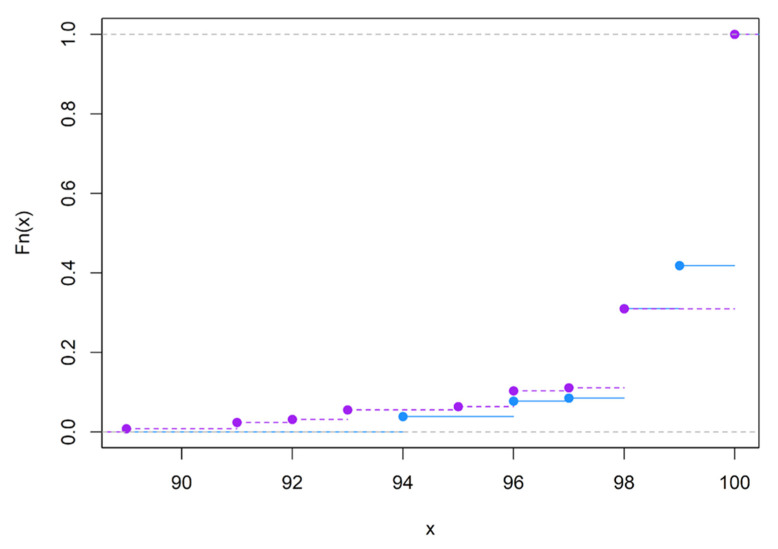
*Empirical cumulative distribution function* (ECDF) of accuracy in the fast block–short SOA per group. *X*-axis refers to the RTs (in m); *y*-axis refers to the ECDF of RTs. Purple = online group. Blue = lab group.

**Figure A11:**
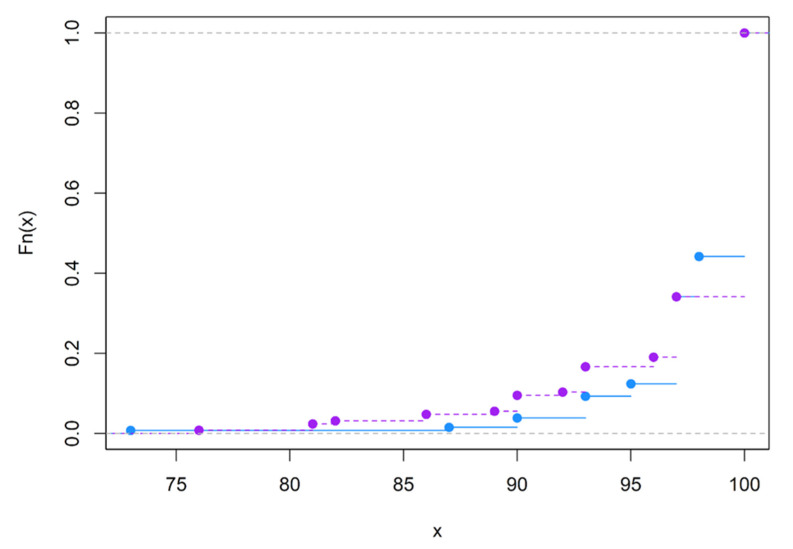
*Empirical cumulative distribution function* (ECDF) of accuracy in the fast block–medium SOA per group. *X*-axis refers to the RTs (in m); *y*-axis refers to the ECDF of RTs. Purple = online group. Blue = lab group.

**Figure A12:**
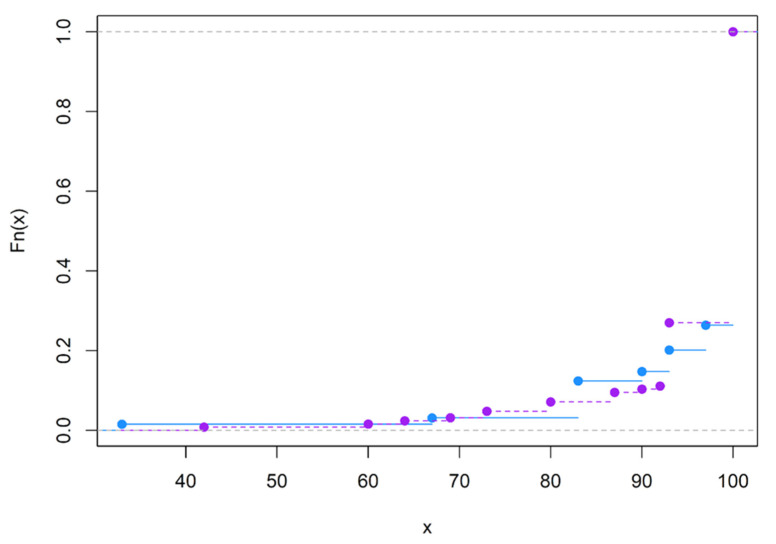
*Empirical cumulative distribution function* (ECDF) of accuracy in the fast block–long SOA per group. *X*-axis refers to the RTs (in m); *y*-axis refers to the ECDF of RTs. Purple = online group. Blue = lab group.

**Figure A13:**
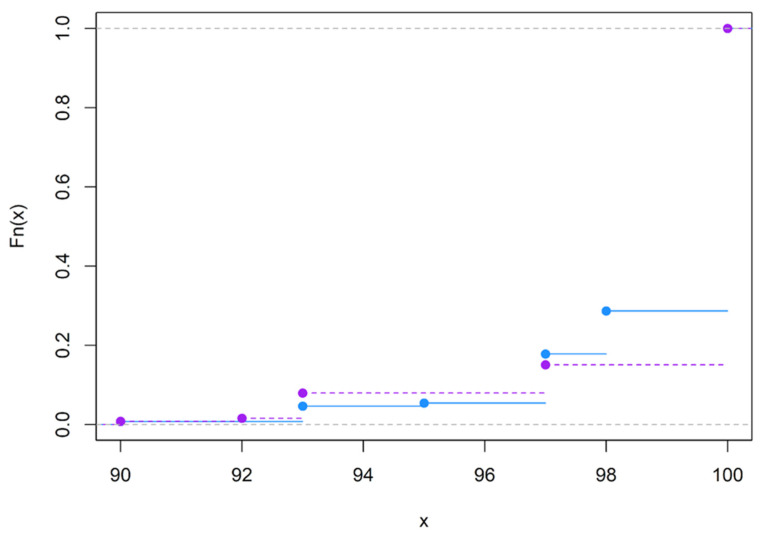
*Empirical cumulative distribution function* (ECDF) of accuracy in the uniform block–short SOA per group. *X*-axis refers to the RTs (in m); *y*-axis refers to the ECDF of RTs. Purple = online group. Blue = lab group.

**Figure A14:**
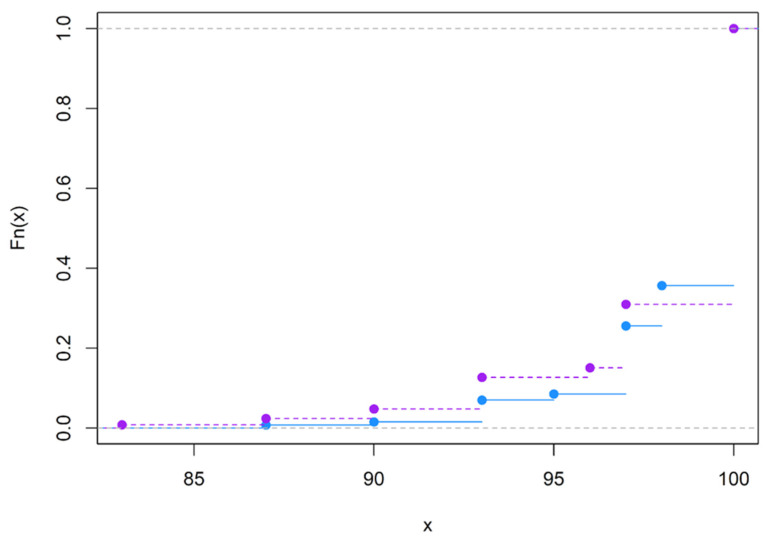
*Empirical cumulative distribution function* (ECDF) of accuracy in the uniform block–medium SOA per group. *X*-axis refers to the RTs (in m); *y*-axis refers to the ECDF of RTs. Purple = online group. Blue = lab group.

**Figure A15:**
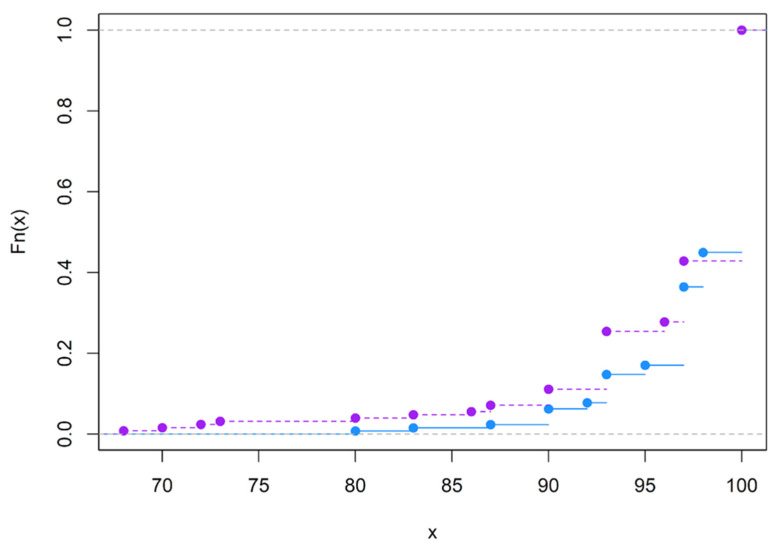
*Empirical cumulative distribution function* (ECDF) of accuracy in the uniform block–long SOA per group. *X*-axis refers to the RTs (in m); *y*-axis refers to the ECDF of RTs. Purple = online group. Blue = lab group.

**Figure A16:**
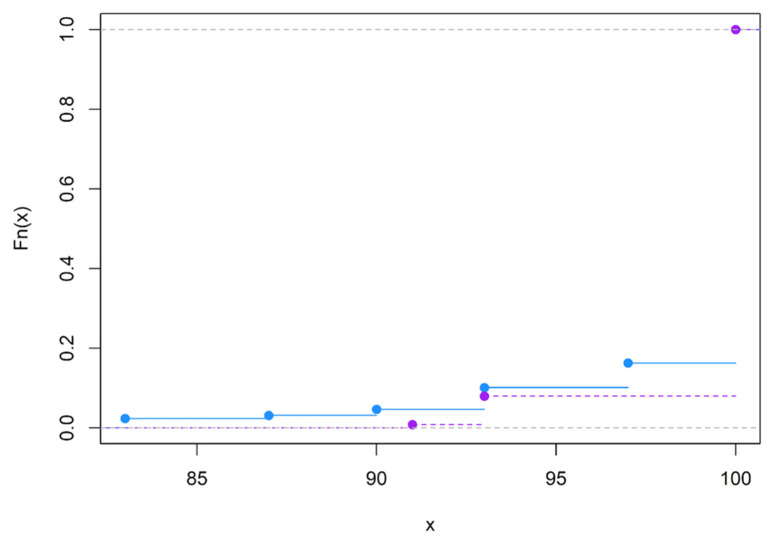
*Empirical cumulative distribution function* (ECDF) of accuracy in the slow block–short SOA per group. *X*-axis refers to the RTs (in m); *y*-axis refers to the ECDF of RTs. Purple = online group. Blue = lab group.

**Figure A17:**
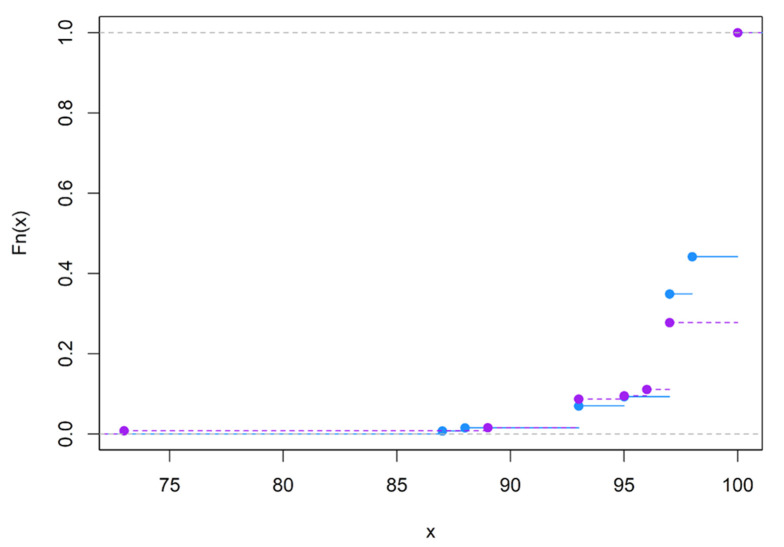
*Empirical cumulative distribution function* (ECDF) of accuracy in the slow block–medium SOA per group. *X*-axis refers to the RTs (in m); *y*-axis refers to the ECDF of RTs. Purple = online group. Blue = lab group.

**Figure A18:**
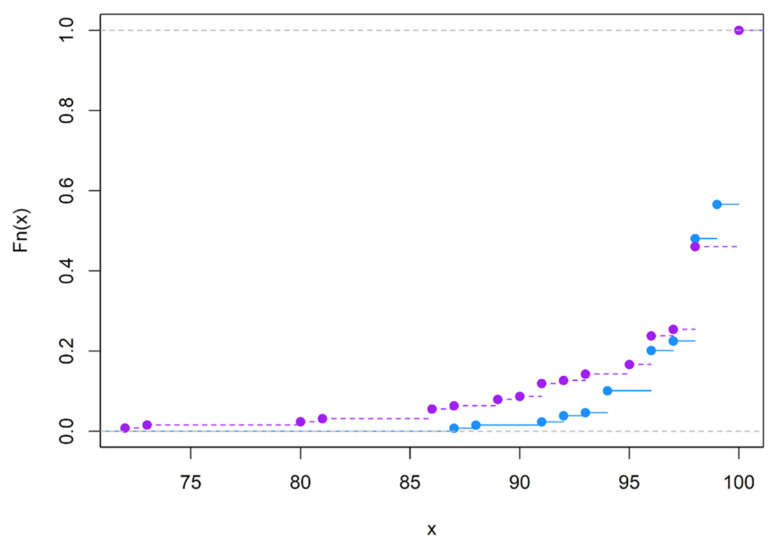
*Empirical cumulative distribution function* (ECDF) of accuracy in the slow block–long SOA per group. *X*-axis refers to the RTs (in m); *y*-axis refers to the ECDF of RTs. Purple = online group. Blue = lab group.

Visual inspection of accuracy ECDF plots (see Appendix B, Figure A10, Figure A11, Figure A12, Figure A13, Figure A14, Figure A15, Figure A16, Figure A17 and Figure A18) revealed a good overlap between the distribution curves of the two groups (online vs. lab) within all the blocks (fast, uniform, slow) and SOA intervals (short, medium, long). Visual inspection’s qualitative analysis is supported by the results of Kolmogorov–Smirnov test comparing accuracy distributions between the two groups: no statistically significant difference was found between the accuracy scores (%) of the two groups in any block and SOA interval (see Table 3).

### 3.3. Statistical Models

#### 3.3.1. Reaction Times

**Figure 2:**
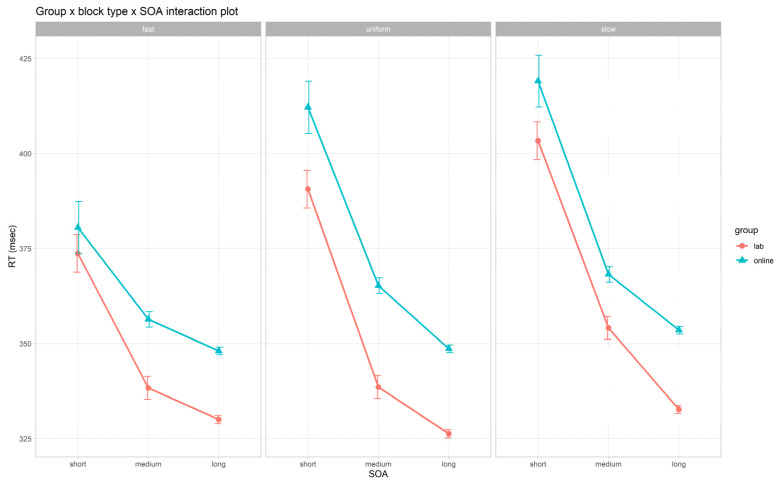
*Group* × *block type* × *SOA* interaction plot on reaction times (RTs, in ms). Bars refer to standard error (*SE*). The log-RT has been converted for graphical purposes. SOA, stimulus onset asynchrony.

The LMM on log-RTs is summarized in Figure 2 and Table 4 and Table S1. We found significant main effects of *group* (*F*(1, 251) = 4.67, *p* = 0.032), *SOA* (*F*(2, 2022) = 580.19, *p* < 0.001), *block type* (*F*(2, 2022) = 38.43, *p* < 0.001), and *age* (*F*(1, 251) = 111.30, *p* < 0.001). With regards to the *group* main effect, as hypothesized (H1a), participants showed significantly slower RTs in the online as compared to the lab setting (lab vs. online: *t*(251) = −2.16, *p* = 0.032). As for the *SOA* main effect, we replicated the attended results (H2a), with increasingly slower RTs from the long to the medium and short SOA (long vs. medium: *t*(2022) = −7.75, *p* < 0.001; long vs. short: *t*(2022) = −32.61, *p* < 0.001; medium vs. short: *t*(2022) = −24.86, *p* < 0.001). Concerning the *block type* main effect, as hypothesized (H2b), we found faster RTs in fast and slower RTs in slow as compared to uniform blocks (fast vs. uniform: *t*(2022) = −4.21, *p* < 0.001; slow vs. uniform: *t*(2022) = 4.55, *p* < 0.001). Lastly, as for the *age* main effect, as hypothesized (H4), we found significantly slower RTs with increasing age (*t*(251) = 10.55, *p* < 0.001).

Moreover, as hypothesized (H3), the LMM showed a significant interaction between *group* and *block type* (*F*(2, 2022) = 5.35, *p* = 0.005): the online group showed significantly slower RTs as compared to the lab group but only in the slow (lab vs. online: *t*(272) = −2.12, *p* = 0.035) and in the uniform blocks (lab vs. online: *t*(272) = −2.68, *p* = 0.008). Interestingly, no significant between-group differences were found within the fast blocks (lab vs. online; *t*(272) = −1.55, *p* = 0.121).

#### 3.3.2. Accuracy

**Figure 3:**
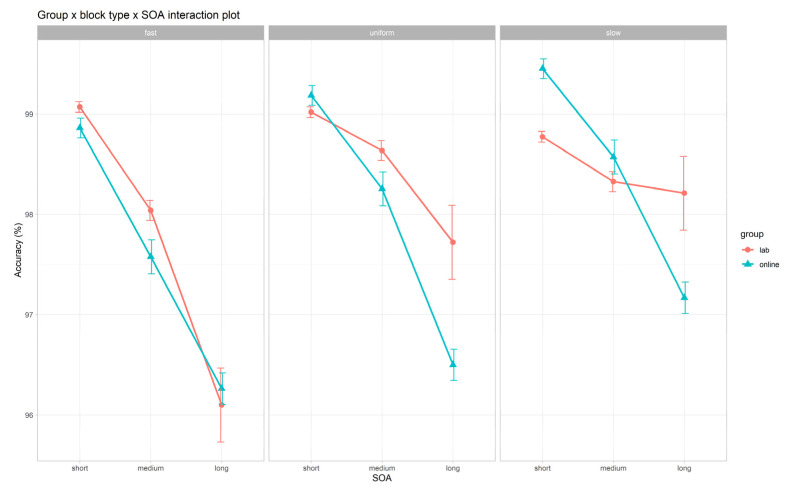
*Group* × *block type* × *SOA* interaction plot on accuracy. Bars refer to standard error (*SE*). SOA, stimulus onset asynchrony.

The GLMM on accuracy is summarized in Figure 3 and Table 5 and Table S2. We found significant main effects of *SOA* (χ^2^(2) = 163.37, *p* < 0.001), *block type* (χ^2^(2) = 20.72, *p* < 0.001), and *gender* (χ^2^(1) = 6.14, *p* = 0.013). As hypothesized (H1b), no significant main effect of the *group* emerged (χ^2^(1) = 0.10, *p* = 0.746). With regards to the *SOA* main effect, we found increasing accuracy from the long to the medium and short SOA (long vs. medium: *z* = −6.05, *p* < 0.001; long vs. short: *z* = −7.38, *p* < 0.001; medium vs. short: *z* = −5.01, *p* < 0.001). Concerning the *block type* main effect, we found a more accurate performance in slow as compared to fast blocks (slow vs. fast: *z* = 3.22, *p* = 0.004) and a less accurate performance in fast as compared to uniform blocks (fast vs. uniform: *z* = −2.35, *p* = 0.049). Lastly, as for the *gender* main effect, we found that female participants (69%) were slightly more accurate than males (male vs. female: *z* = −2.48, *p* = 0.013).

Moreover, we found significant interactions between *group* and *SOA* (χ^2^(2) = 9.15, *p* = 0.010) and between *group*, *SOA,* and *block type* (χ^2^(4) = 10.90, *p* = 0.028). However, the only significant post hoc contrast was found between the online and lab settings within short SOA intervals regardless of block (short SOA: lab vs. online: *z* = −2.32, *p* = 0.021), suggesting a slightly more accurate performance in the online setting.

#### 3.3.3. Delta Scores

**Figure 4:**
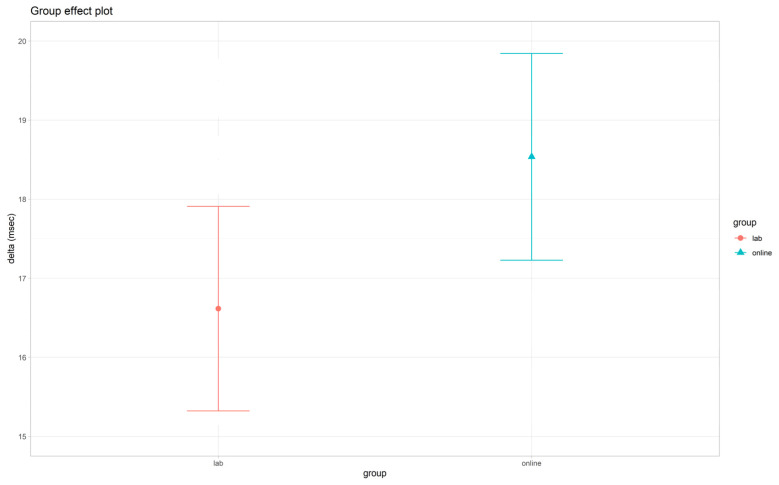
*Group* effect plot on delta scores (difference in RTs between slow and fast blocks). Bars refer to standard error (*SE*).

The LM on delta scores is summarized in Figure 4 and Table 6 and Table S3. Interestingly, as hypothesized (H2c), in both the groups, mean delta scores were positive. We found a significant main effect of *age* (*F*(1, 2289) = 138.5, *p* < 0.001), with greater delta scores with increasing age, suggesting a less efficient implicit adaptation of motor response to between-blocks task speed changes in older participants. As hypothesized (H3c), the *group* did not exert a significant modulation on delta scores (*F*(1, 2289) = 1.08, *p* = 0.298), thus suggesting that the implicit modulation of RTs as a function of task changes in the global predictive context occurred in a comparable way in the two settings.

## 4. Discussion

The present work represents to the best of our knowledge the first attempt to compare behavioral data collected across the adult lifespan in the traditional laboratory setting with ones collected in an online setting by employing a task inducing a modulation of participants’ alerting at an *implicit* level (i.e., DTP task).

As for the setting effect, we confirmed the expected results of a significant delay (here, of about 20 ms) in response speed (see H1a), not implying accuracy differences though (see H1b), in the online setting. This is consistent with recent literature suggesting that RTs are systematically delayed (usually within a range of 25–60 ms) in online experiments [22,24,25,26], and it can be explained by the inevitable technical variability in browsers/operating systems within participants’ devices [3,22].

Moreover, as hypothesized, we replicated in the online setting and across the whole age range considered (19–69 years) all the task-specific experimental effects already found in the lab (and described in [34]): (i) faster RTs and lower accuracy in trials with long vs. medium and short SOA (see H2a); (ii) faster RTs in fast blocks and slower RTs in slow blocks as compared to the uniform block (see H2b); and (iii) the implicit learning effect, as reflected by a positive delta score (of about 16 ms for the lab and 18 ms for the online setting) between slow and fast blocks (see H2c).

Furthermore, age showed the expected modulation on response speed (see H4), with progressively slower RTs with increasing age. Although a thorough interpretation of age-related effects on task performance goes beyond the aims of this study, it is interesting to note that as net of the RTs slow down, older participants showed a less efficient implicit adaptation of their motor response to the task-induced between-blocks speed changes (as reflected by greater delta scores). A similar finding was reported for younger vs. older children by [34] in their original study. Taken together, the evidence that both younger children and older adults exhibit less efficient implicit motor adaptation to the global, block-wise changes in task speed, which may reflect age-related strategic adjustment of proactive motor control. More specifically, we may speculate that the low processing speed (i.e., overall slower RTs) observed in the early and late stages of the human lifespan may provide more space for behavioral advantage induced by implicit learning. In other words, people who have slow processing speed (i.e., younger children and older adults) may benefit more from implicit experimental manipulations since they have greater psychomotor gain margin (high delta score). By contrast, people who show fast processing speed (i.e., older children, adolescents, and young adults) have already quasi-ceiling behavioral performance. Hence, they will generally benefit less from experimental manipulations implying motor adjustments (low delta score). However, the investigation of age effects on implicit flexibility is beyond the scope of the present study and is currently under investigation by our group (Mento et al., in preparation).

Crucial for the scope of the present study, our results suggested that, regardless of age and sex, the implicit motor adaptation occurred similarly in the online and lab settings since no significant differences in delta scores emerged between them. Participants in the online setting seem therefore able to implicitly infer the task temporal structure and to proactively adapt their response speed depending on global predictive rules, similarly to the way it occurs when the DTP task is administered in the lab. Thus, consistently with the literature [23,27,28,29,30], our results provide evidence that both the direction and magnitude of the DTP task-specific effects are comparable between the online and laboratory settings.

Lastly and most interestingly, some interactions between the setting and DTP task’s specific features emerged, as hypothesized (see H3). More in detail, we found that participants in the online setting showed a significantly slower response speed in slow and uniform blocks (but not in fast blocks) and a slightly more accurate performance in trials with short SOA intervals (but not in trials with medium or long SOA) as compared to participants in the lab. These interactions clearly revealed how task-specific behavioral features ascribable to participants’ alerting state may be further modulated by the task administration setting, with experimental conditions being differently affected depending on the response rate they implicitly induce. In fact, at a global level, the systematic delay in response speed expected in the online setting emerged only in those task blocks involving a slower response rate (i.e., slow and uniform) and thus a potential decrease of participants’ alerting. On the contrary, no delay emerged in blocks inducing a faster response rate (i.e., fast) since the higher stimuli frequency may have pushed participants towards a heightened alerting state, which in turn may have resulted in a faster performance eventually compensating for the RTs delay. The different arousal levels induced by the task thus interacted with the online setting, leading participants to a heightened vulnerability to distractions and attentional shifts (which are *per se* greater and less controllable online as compared to the lab setting) [17,58,59], especially in those experimental conditions implying a less urgent response rhythm. At a local level, instead, conditions implying a faster response rate (i.e., short SOA intervals), which elicited a better overall performance in both settings, underwent a slight (0.2%) but significant accuracy increase in the online setting. It may be possible that a potential increment of participants’ alerting, as induced by a local predictive rule implying a faster response rate, may have supported heightened attention and response control, eventually leading to a more accurate performance. Thus, in summary, participants’ behavioral performance (as reflected by both response speed and accuracy) seems to be more sensitive to task-induced alerting shifts in the online as compared to the lab setting, leading to either a heightened or reduced efficiency depending on a faster or slower response rate of experimental conditions, respectively. This may depend on the inevitably less strict time-by-time control of participants’ performance typical of the online setting [60,61].

As a limit of the present work worth expanding on, since our experimental design did not allow us to distinguish whether the interactions between the setting and task’s specific features were exclusively associated with the DTP task or whether they may be shared with other implicit tasks, we encourage future research to implement new online vs. lab comparison studies specifically targeting implicit tasks. As another potential limitation of this work, it is worth noting that a different software has been used for lab and online data collection (E-Prime vs. OpenSesame, respectively). However, both software types allow for a millisecond precision timing in stimulus presentation; thus, any slight difference can be reasonably considered of negligible significance and addressed to the specific effect of the setting rather than to software differences.

## 5. Conclusions

In summary, our results support our hypotheses, and they contribute in advancing knowledge on the interactions between data collection setting (online vs. lab) and task-specific features. This work integrates well with existing studies suggesting that online data collection may represent a methodologically sound tool for experimental psychological research [3,20,31,32]. In fact, the online setting proved to be effective in replicating the attended experimental effects not only when the task implies a fine stimulus/response timing (as already demonstrated by the literature) [22,62] but also when this fine timing is induced at an implicit level (as we demonstrated in the present work with the DTP task). However, our results suggest not negligible caution in the case of tasks inducing different response rates between conditions. In fact, we collected evidence that the online setting is particularly sensitive to task-specific implicit alerting shifts, eventually leading to a less efficient performance in experimental conditions with a less urgent response rate. This may introduce a biasing factor threatening the methodological soundness of the online version of the task, which must be taken into careful account. We thus suggest, as potential countermeasures, to provide online tasks with clear and simple instructions, short breaks during the task, and a reasonable overall duration. We also suggest employing experimental tasks with a fixed temporal structure and fast inter-stimulus intervals in order to maintain high and constant alerting levels and further facilitate participants’ attention and motivation. Introducing trial- or block-wise performance feedback throughout the task may be a useful additional countermeasure, too.

From a more general point of view, beyond the specific results reported here, this article opens up interesting food for thought about the opportunity to use (or not) online data collection methodology in a systematic way in psychological research. On the one hand, it is important to consider that our data refer to a particular task and have made it possible to answer a very specific question. Therefore, it is difficult for us to draw general and definitive conclusions. On the other hand, the fact that our results confirm previous studies on the reliability of this approach could lead us to evaluate the opportunity of use it for any experimental circumstance. However, it should be borne in mind that online research, although a potentially very valid ally of every researcher in the psychological field, inevitably involves an increase in the variability (and therefore in the noise) of the data collected. Therefore, its use could be more appropriate within experimental paradigms that promise experimental effects able to survive a greater intra and inter individual variability. Conversely, online collection may be less advantageous in cases of extremely subtle effects that require high control of the experimental setting. Consequently, it is of fundamental importance to evaluate on a case-by-case basis whether to resort to this alternative or to follow the more traditional, old path of controlled laboratory research. However, a thorough examination of all cases where the advantages of online research outweigh the potential disadvantages is beyond the scope of this paper. Therefore, a systematic comparison within the same study between these two methods using different experimental tasks with effects of different magnitudes and possibly in multiple fields of psychological research is still as yet missing as appropriate and welcome in psychological research literature.

## Figures and Tables

**Table 1:** Main demographic characteristics (age and gender) of the two groups of participants (online vs. lab). Mean (*M*) age, standard deviation (*SD*), age range, and gender for online and lab groups are reported.

Group	Gender	N	*M* ± *SD* (Range)	Group *M* ± *SD* (Range)
Online	M	34	50.14 ± 17.14 (20–69)	40.80 ± 17.75 (19–69)
	F	92	37.35 ± 16.70 (19–69)	
Lab	M	44	49.39 ± 15.14 (22–69)	40.55 ± 17.65 (19–69)
	F	85	35.97 ± 17.12 (19–69)	

**Table 2:** Descriptive statistics of online and lab groups. Mean (*M*) and standard deviation (*SD*) of reaction times (RT, in ms) and accuracy (Acc, in percentage) are reported for each group (online vs. lab) and experimental condition (fast vs. uniform vs. slow block type × short vs. medium vs. long SOA—stimulus onset asynchrony). Delta scores (in ms) are reported for each group (online vs. lab).

Group	Block	SOA						Delta
		Short		Medium		Long		
		RT (m)	Acc (%)	RT (m)	Acc (%)	RT (m)	Acc (%)	
		*M* ± *SD*	*M* ± *SD*	*M* ± *SD*	*M* ± *SD*	*M* ± *SD*	*M* ± *SD*	*M* ± *SD*
Online	Fast	380.5 ± 99.5	98.9 ± 2.2	356.4 ± 101.2	97.6 ± 4.5	348.0 ± 93.2	96.3 ± 8.8	−18.64 ± 32.1
	Uniform	412.2 ± 108.2	99.2 ± 2.1	365.2 ± 105.4	98.3 ± 3.2	348.7 ± 95.0	96.5 ± 6.1	
	Slow	419.0 ± 102.5	99.5 ± 1.9	368.2 ± 104.6	98.6 ± 3.8	353.6 ± 99.5	97.2 ± 5.1	
Lab	Fast	373.7 ± 115.0	99.1 ± 1.4	338.3 ± 101.2	98.0 ± 3.4	330.0 ± 98.0	96.1 ± 10.2	−16.52 ± 55.5
	Uniform	390.7 ± 134.6	99.0 ± 1.9	338.6 ± 113.3	98.6 ± 2.3	326.3 ± 105.3	97.7 ± 3.6	
	Slow	403.4 ± 143.7	98.8 ± 3.4	354.1 ± 119.4	98.3 ± 2.4	332.7 ± 108.7	98.2 ± 2.4	

**Table 3:** Online and lab reaction times (RT, in ms) and accuracy (Acc, in percentage) distributions comparison using Kolmogorov–Smirnov test. Significance level is set to <0.05. Bold *p*-values (*p*) signal conditions in which online and lab distributions do not significantly overlap. While between-group accuracy distributions revealed a comparable overlap across all experimental conditions, between-group RT distributions showed an overlap in the fast block and only a partial overlap in the uniform and slow blocks.

Condition		RT (m)		Acc (%)	
Block	SOA	Kolmogorov–Smirnov Test	*p*	Kolmogorov–Smirnov Test	*p*
Fast	Short	D = 0.146	0.134	D = 0.109	0.434
	Medium	D = 0.127	0.254	D = 0.101	0.539
	Long	D = 0.167	0.057	D = 0.068	0.928
Uniform	Short	D = 0.209	**0.008**	D = 0.136	0.189
	Medium	D = 0.230	**0.002**	D = 0.066	0.947
	Long	D = 0.245	**0.000**	D = 0.107	0.456
Slow	Short	D = 0.194	**0.017**	D = 0.083	0.767
	Medium	D = 0.185	**0.025**	D = 0.164	0.065
	Long	D = 0.193	**0.018**	D = 0.106	0.476

**Table 4:** Main results of the linear mixed-effects model (LMM) on log-transformed reaction times (RTs), namely *F*-test (*F*), degrees of freedom (*df*), and *p*-values (*p*), are reported. Bold *p*-values signal statistical significance.

Predictors	*F*	*df*	*p*
SOA	580.19	2, 2022	**<0.001**
Block	38.43	2, 2022	**<0.001**
Group	4.67	1, 251	**0.032**
Gender	3.20	1, 251	0.075
Age	111.30	1, 251	**<0.001**
SOA × Block	13.59	4, 2022	**<0.001**
SOA × Group	1.29	2, 2022	0.276
Block × Group	5.35	2, 2022	**0.005**
SOA × Block × Group	1.01	4, 2022	0.403

**Table 5:** Main results of the generalized linear mixed-effects model (GLMM) on accuracy, namely *chi-square* test (χ^2^), degrees of freedom (*df*), and *p*-values (*p*), are reported. Bold *p*-values signal statistical significance.

Predictors	χ^2^	*df*	*p*
SOA	163.37	2	**<0.001**
Block	20.72	2	**<0.001**
Group	0.10	1	0.746
Gender	6.14	1	**0.013**
Age	0.61	1	0.434
SOA × Block	3.87	4	0.424
SOA × Group	9.15	2	**0.010**
Block × Group	1.00	2	0.607
SOA × Block × Group	10.90	4	**0.028**

**Table 6:** Main results of the linear model (LM) on delta scores, namely *F*-test (*F*), degrees of freedom (*df*), and *p*-values (*p*), are reported. Bold *p*-values signal statistical significance.

Predictors	*F*	*df*	*p*
Group	1.08	1, 2289	0.298
Gender	0.06	1, 2289	0.812
Age	138.50	1, 2289	**<0.001**

## Data Availability

All the data and analysis code reported in the present manuscript are available in the OSF repository https://osf.io/m8wjb/?view_only=5de9247d0ef248448b86ef364e95580f, accessed on 7 July 2022.

## Supplementary Materials

The following supporting information can be downloaded at: https://www.mdpi.com/article/10.3390/brainsci12081061/s1, Table S1: Fixed and random effects resulting from the linear mixed-effects model (LMM) on the log-transformed reaction times (log-RT): estimates (in logit scale), standard error (*SE*), 95% confidence interval (*CI*), statistics (*t*-value), *p*-values (*p*), and degrees of freedom (*df*) are reported. Bold *p*-values signal statistical significance. The marginal and conditional R2 are also reported. SOA, stimulus onset asynchrony; Table S2: Fixed and random effects resulting from the generalized linear mixed-effects model (GLMM) on accuracy: estimates (in odds ratios), standard error (*SE*), 95% confidence interval (*CI*), statistics (*z*-test), *p*-values (*p*), and degrees of freedom (*df*) are reported. Bold *p*-values signal statistical significance. The marginal and conditional R2 are also reported. SOA, stimulus onset asynchrony; Table S3: Results from the linear model (LM) on Delta scores: estimates (in m), standard error (*SE*), 95% confidence interval (*CI*), statistics (*t*-test), *p*-values (*p*), and degrees of freedom (*df*) are reported. Bold *p*-values signal statistical significance. The R2 and the adjusted R2 are also reported.

## Appendix A

Empirical cumulative distribution function (ECDF) of RTs.

## Appendix B

Empirical cumulative distribution function (ECDF) of accuracy.
