# Supplementary material for: Doing Experimental Psychological Research from Remote: How Alerting Differently Impacts Online vs. Lab Setting
Source: Brain Sci. 2022 Aug 10;12(8):1061. doi: 10.3390/brainsci12081061 (PMC9405885; doi:10.3390/brainsci12081061)
Supplement: Supplementary file 1 [file brainsci-12-01061-s001.zip › brainsci-1832096-supplementary.pdf]

SUPPLEMENTARY MATERIAL

Doing Experimental Psychological Research from Remote: How Alerting Differently Impacts Online vs. Lab Setting

Fiorella Del Popolo Cristaldi, Umberto Granziol, Irene Bariletti, Giovanni Mento

Table S1. Fixed and random effects resulting from the Linear Mixed-effects Model (LMM) on the log-transformed reaction times (log-RT): estimates (in logit scale), standard error (*SE*), 95% confidence interval (*CI*), statistics (*t*-value), *p*-values (*p*) and degrees of freedom (*df*) are reported. Bold *p*-values signal statistical significance. The marginal and conditional R^2 are also reported. SOA = Stimulus Onset Asynchrony

	log-RT					
Predictors	***Estimates***	***SE***	***CI***	***Statistic***	***p***	***df***
(Intercept)	5.45	0.04	5.37 – 5.52	144.62	**<0.001**	2271.00
SOA [medium]	0.05	0.01	0.03 – 0.06	6.03	**<0.001**	2271.00
SOA [short]	0.18	0.01	0.16 – 0.20	22.31	**<0.001**	2271.00
block [fast]	-0.01	0.01	-0.02 – 0.01	-0.86	0.388	2271.00
block [uniform]	-0.01	0.01	-0.03 – 0.00	-1.79	0.074	2271.00
group [lab]	-0.07	0.03	-0.12 – -0.01	-2.29	**0.022**	2271.00
gender [M]	-0.05	0.03	-0.11 – 0.01	-1.79	0.074	2271.00
age years	0.01	0.00	0.01 – 0.01	10.55	**<0.001**	2271.00
SOA [medium] * block [fast]	-0.03	0.01	-0.05 – -0.00	-2.33	**0.020**	2271.00
SOA [short] * block [fast]	-0.08	0.01	-0.10 – -0.05	-6.57	**<0.001**	2271.00
SOA [medium] * block [uniform]	-0.01	0.01	-0.03 – 0.01	-0.96	0.338	2271.00
SOA [short] * block [uniform]	-0.01	0.01	-0.03 – 0.01	-0.83	0.409	2271.00
SOA [medium] * group [lab]	0.02	0.02	-0.01 – 0.05	1.11	0.269	2271.00
SOA [short] * group [lab]	0.01	0.02	-0.02 – 0.04	0.45	0.655	2271.00
block [fast] * group [lab]	0.01	0.02	-0.02 – 0.04	0.74	0.458	2271.00
block [uniform] * group [lab]	-0.01	0.02	-0.04 – 0.03	-0.31	0.758	2271.00
SOA [medium] * block [fast] * group [lab]	-0.01	0.02	-0.06 – 0.03	-0.61	0.540	2271.00
SOA [short] * block [fast] * group [lab]	0.02	0.02	-0.02 – 0.07	1.04	0.299	2271.00
SOA [medium] * block [uniform] * group [lab]	-0.03	0.02	-0.07 – 0.02	-1.25	0.210	2271.00
SOA [short] * block [uniform]) * group [lab]	-0.00	0.02	-0.05 – 0.04	-0.10	0.923	2271.00

Random Effects

σ^2	0.01
$\tau_{00\ n}$	0.04
ICC	0.84
N n	255
Observations	2293
Marginal R^2 / Conditional R^2	0.332 / 0.893

Table S2. Fixed and random effects resulting from the Generalized Linear Mixed-effects Model (GLMM) on accuracy: estimates (in odds ratios), standard error (*SE*), 95% confidence interval (*CI*), statistics (*z*-test), *p*-values (*p*) and degrees of freedom (*df*) are reported. Bold *p*-values signal statistical significance. The marginal and conditional R^2 are also reported. SOA = Stimulus Onset Asynchrony

			accuracy		
Predictors	***Odds Ratios***	***SE***	***CI***	***Statistic***	***p***
(Intercept)	58.93	13.06	38.18 – 90.98	18.40	**<0.001**
SOA [medium]	1.48	0.16	1.19 – 1.84	3.51	**<0.001**
SOA [short]	7.35	4.00	2.53 – 21.37	3.66	**<0.001**
block [fast]	0.70	0.11	0.51 – 0.97	-2.16	**0.031**
block [uniform]	0.78	0.07	0.65 – 0.94	-2.63	**0.008**
group [lab]	1.19	0.22	0.82 – 1.71	0.92	0.359
gender [M]	0.65	0.11	0.46 – 0.91	-2.48	**0.013**
age years	1.00	0.00	0.99 – 1.01	0.78	0.434
SOA [medium] * block [fast]	1.00	0.20	0.67 – 1.49	-0.02	0.988
SOA [short] * block [fast]	0.44	0.25	0.14 – 1.34	-1.45	0.147
SOA [medium] * block [uniform]	1.30	0.21	0.94 – 1.78	1.60	0.110
SOA [short] * block [uniform]	0.45	0.25	0.15 – 1.34	-1.44	0.151
SOA [medium] * group [lab]	0.54	0.12	0.35 – 0.83	-2.81	**0.005**
SOA [short] * group [lab]	0.11	0.12	0.01 – 0.92	-2.03	**0.042**
block [fast] * group [lab]	0.52	0.17	0.28 – 0.98	-2.01	**0.044**
block [uniform] * group [lab]	1.04	0.19	0.73 – 1.50	0.23	0.819
SOA [medium] * block [fast] * group [lab]	2.90	1.18	1.31 – 6.46	2.61	**0.009**
SOA [short] * block [fast] * group [lab]	11.97	13.69	1.27 – 112.58	2.17	**0.030**
SOA [medium] * block [uniform] * group [lab]	1.52	0.49	0.80 – 2.87	1.28	0.199
SOA [short] * block [uniform] * group [lab]	4.38	4.93	0.48 – 39.75	1.31	0.190

Random Effects

σ^2	3.29
$\tau_{00\ n}$	1.09
ICC	0.01
N n	255
Observations	68850
Marginal R^2 / Conditional R^2	0.163 / 0.173

Table S3. Results from the Linear Model (LM) on Delta scores: estimates (in msec), standard error (*SE*), 95% confidence interval (*CI*), statistics (*t*-test), *p*-values (*p*) and degrees of freedom (*df*) are reported. Bold *p*-values signal statistical significance. The R^2 and the adjusted R^2 are also reported.

	Delta					
Predictors	***Estimates***	***SE***	***CI***	***Statistic***	***p***	***df***
(Intercept)	-3.96	12.73	-29.03 – 21.11	-0.31	0.756	251.00
group [lab]	-1.33	9.07	-19.20 – 16.54	-0.15	0.884	251.00
gender [M]	-11.10	10.46	-31.71 – 9.50	-1.06	0.290	251.00
age years	0.96	0.27	0.43 – 1.50	3.55	**<0.001**	251.00
Observations	255					
R^2 / R^2 adjusted	0.048 / 0.037					
